# Effectiveness of a Mobile Breastfeeding Monitoring Tool Among Mothers in WeChat Groups on Breastfeeding Exclusivity and Self-Efficacy: Intention-to-Treat and Per-Protocol Analyses of a Randomized Controlled Trial

**DOI:** 10.2196/67024

**Published:** 2025-08-15

**Authors:** Ni Jia, Jean J Schensul, Meixian Zhang, Lianfang Kong, Qi Yan, Yaohua Dai

**Affiliations:** 1 Capital Center for Children's Health, Capital Medical University, Capital Institute of Pediatrics Beijing China; 2 Institute for Community Research Hartford, CT United States; 3 Evidence-based Medicine Center Taizhou Hospital of Zhejiang Province affiliated to Wenzhou Medical University Taizhou China; 4 Department of Early Child Development Haidian Maternal and Child Health Hospital Beijing China

**Keywords:** mHealth, exclusive breastfeeding, predominant breastfeeding, breastfeeding self-efficacy, per-protocol analysis, mental health

## Abstract

**Background:**

Globally, the pursuit of exclusive breastfeeding (EBF) remains a formidable challenge. The surge in popularity of mobile health interventions demonstrates its potential as a promising avenue for promoting breastfeeding practices. Nevertheless, research investigating breastfeeding monitoring interventions via mobile health remains scarce among diverse populations.

**Objective:**

This study aimed to use an app called Breastfeeding Aiding Tool to monitor breastfeeding and provide tailored feedback to improve EBF, maternal breastfeeding self-efficacy, and depression status.

**Methods:**

In an unblinded randomized controlled trial, the intervention leveraged the app to monitor breastfeeding practices recorded by users and to provide automatic feedback, facilitating non–face-to-face education and consultation in a WeChat group by 3 health care workers. Mothers in the control group received the same education and consultation services via the WeChat group. Lactating mothers and their healthy primiparous infants aged 35 to 49 days were recruited from 2 clinics, and the online follow-up period was 2 months. Information about breastfeeding practices, maternal breastfeeding confidence, and maternal depression status were collected through SoJump.

**Results:**

From September 2022 to January 2024, a total of 141 eligible mother-infant dyads were recruited offline and allocated to the two groups; 109 (n=55, 50.5% in the intervention group and n=54, 49.5% in the control group) dyads completed the 2-month online follow-up and were included in the analysis, among which 27 (49%) mothers actively engaged with the app. In the per-protocol analysis sample, the rate of EBF of the app-using group was 57% (16/28), compared to 48% (39/81) in the comparison group (*P*=.41; odds ratio [OR] 1.44, 95% CI 0.60-3.41; adjusted OR 1.66, 95% CI 0.59-4.68). The rate of full breastfeeding (comprising predominant breastfeeding and EBF) was significantly higher in the mothers using the app than in the nonusers (26/28, 93% vs 59/81, 73%; *P*=.03; OR 4.85, 95% CI 1.06-22.15; adjusted OR 5.55, 95% CI 1.07-28.83). Maternal breastfeeding self-efficacy in the app-using group improved by an average of 1.36 (95% CI –3.79 to 1.50), while it declined slightly, by 0.16 (95% CI –3.16 to 2.84), in the nonusing group; the difference between the 2 groups was not significant (*P*=.61). The depression scores in the app-using group decreased by an average of 2.29 (95% CI –5.19 to 0.62), whereas they increased by 1.07 (95% CI –0.58 to 2.73) in the nonusing group; there was a significant difference between the two groups (*P*=.046).

**Conclusions:**

Our findings underscore the significant potential of the Breastfeeding Aiding Tool app as an aid to breastfeeding guidance in sustaining breastfeeding practices, reducing formula use, and enhancing positive maternal breastfeeding emotions. To further validate the effectiveness of improving EBF and self-efficacy, studies should aim to address barriers to the app’s acceptability and enroll a larger cohort of mothers.

**Trial Registration:**

Chinese Clinical Trial Registry ChiCTR2200065220; https://www.chictr.org.cn/showprojEN.html?proj=182939

## Introduction

### Background

Breast milk is the optimal food for infants in the first few months, and infants can be exclusively breastfed for 6 or more months [1,2]. The World Health Organization (WHO) recommends 6 months of exclusive breastfeeding (EBF) [3]. Despite the extensive evidence supporting the health and cognitive benefits of breastfeeding for babies, the vast majority of children globally are not breastfed in line with the recommendations [4,5]. Globally, 44% of infants were on EBF for 6 months and only 29.2% in China [6]. One of the goals of the China Children’s Development Plan (2021 to 2030) was to achieve an EBF rate of >50% for infants aged 0 to 6 months [7]. Reaching this goal requires more effective measures to tackle the barriers to promoting EBF in China.

Breastfeeding is affected by a wide range of historical, socioeconomic, cultural, and individual factors, and nonattainment of EBF is also a multifactorial issue [8-10]. Researchers have advocated comprehensive breastfeeding consultation and education initiatives to tackle the barriers to improve breastfeeding knowledge and enhance breastfeeding self-efficacy to promote EBF [11-14]. One approach with promise for addressing these issues is mobile health (mHealth). In recent years, mHealth has dramatically increased in popularity, fueled by its unparalleled flexibility in terms of time and space use, enhanced accessibility, and reduced costs. Health care workers and researchers have hailed it as an efficacious technological intervention for online education and support to foster breastfeeding practices, underscoring its potential to revolutionize health care delivery and support mothers worldwide [15-18].

However, these online interventions focused predominantly on education. To enhance their effectiveness, a multifaceted approach was suggested to integrate online educational materials with interactivity and personalize the intervention content [19,20]. Almohanna et al [20] reviewed the effectiveness of a range of breastfeeding interventions using electronic technologies and concluded that breastfeeding trackers appeared to be the least effective strategy. However, that conclusion undervalued the potential power of the monitoring app as a tool to facilitate education and consultation and to assist mothers with breastfeeding. Dinour and Pole [21] evaluated the features of existing breastfeeding apps and found that those with tracking features were rated more highly by users. Ahmed et al [22,23] conducted interventions that combined interactive web-based breastfeeding monitoring with standard breastfeeding education and concluded that this approach could improve breastfeeding exclusivity, intensity, and duration, as well as promote maternal breastfeeding self-efficacy. The evidence demonstrated that interventions incorporating breastfeeding monitoring, which were readily accepted by mothers, could be an effective approach to enhancing breastfeeding practices. Nevertheless, Dinour [24] pointed out that these apps are predominantly designed with White mothers and infants in mind and, given this narrow focus, emphasized the necessity for further research to ensure that they can be effectively used by a more diverse range of populations.

### Objectives

Despite the abundance of software with tracking capabilities currently available on the market, there are significant concerns regarding the inadequate coverage of information provided by these apps, as well as the prevalence of incorrect or incomplete data [25,26]. Moreover, the overwhelming influx of health-related information on social media platforms has led to a phenomenon known as online health information anxiety. This issue necessitates the intervention and improvement efforts from specialists [27]. To the best of our knowledge, there have been no trials in China reported to evaluate the effectiveness of a smartphone app that monitored breastfeeding and provided feedback to maintain EBF.

On the aforementioned basis, we developed an app called Breastfeeding Aiding Tool and conducted a study aimed to use the app to monitor breastfeeding and provide tailored feedback as a non–face-to-face intervention among mothers receiving breastfeeding education and consultation via a WeChat (Tencent Holdings Limited) group to improve EBF, maternal breastfeeding self-efficacy, and depression status in urban China.

## Methods

### Study Design and Sample Size

A cluster randomized controlled trial was conducted with an intervention group (app and WeChat group) and a control (WeChat group only) group. Intervention and follow-up were conducted using smartphones. The intervention study was registered in the Chinese Clinical Trial Registry (ChiCTR2200065220).

A total initial sample size of 126 (n=63, 50% in each group) mother-infant dyads was estimated by G*Power (version 3.1; Heinrich Heine University) based on an 85% statistical power at a 2-tailed α of .05. The breastfeeding rates of intervention and control groups were estimated as 60% and 30%, respectively, with the attrition rate estimated as 20%. All rates were estimated based on our pilot study.

### Recruitment

Participants were recruited from health care clinics of 2 comparable maternal and child health care hospitals in urban areas of Beijing and screened by child health care providers who were part of the research team. The inclusion and exclusion criteria are provided in Textbox 1.

**Inclusion and exclusion criteria.**

**Inclusion criteria**

- Infants aged 35 to 49 days, with gestational age ≥37 weeks, birth weight ≥2500 g, and Apgar score ≥9
- Infants without serious health problems, including congenital and infectious diseases (eg, tetralogy of Fallot and HIV infection)
- Infants without sucking and swallowing problems
- First-time mothers
- Mothers who breastfed their infants in the lactogenesis stage and intended to fully or exclusively breastfeed before complementary feeding

**Exclusion criteria**

- Multiparous women
- Infants and mothers with serious diseases, hampering breastfeeding

### Randomization and Blinding

The recruitment process for this study was conducted in 2 comparable hospitals, which were frequented by mothers bringing their children for routine well-baby checkups. These clinics served as the primary venues for enrolling both the treatment and control group dyads. A cluster randomized method was used at the onset of the recruitment period. Specifically, the visit days in each clinic were randomly designated as either “treatment-1” days or “control-2” days. On each assigned visit day (either 1 or 2), it was anticipated that 1 to 3 dyads would be recruited, continuing until the required sample size for each group was achieved. All mother-infant dyads who met the established recruitment criteria were selected and enrolled in the study. However, due to the unpredictable number of eligible dyads attending the clinic on a daily basis, the actual recruitment pattern varied. Over the entire recruitment period spanning 96 days, 1 to 3 dyads were recruited on 85 days, while 4 to 8 dyads were recruited on the remaining 11 days.

A research designer generated the random allocation sequence. Health care providers in the clinics assigned participants to the intervention and control groups according to the allocated numbers on the recruiting days.

On the basis of the informed consent procedures, participants knew whether they were in the intervention or control group. Thus, blinding was not performed due to the nature of the intervention [28].

### Protocol Deviations

#### Type of Randomized Controlled Trial

Initially, we had planned to use parallel randomization, enrolling mother-infant dyads individually. However, we soon encountered a challenge. Mothers often arrived at the clinics in groups on the same day and were acquainted with one another. This familiarity compromised the effectiveness of individual randomization. Mothers in the control group would learn about the intervention from their friends in the treatment group and express a desire to join, leading to potential contamination or instability in group membership. To address this issue and prevent contamination or crossover between groups, we decided to recruit treatment and control dyads on separate days, adopting the cluster randomized design described in the Randomization and Blinding section.

#### Sample Size Re-Estimation

A total initial sample size of 280 (n=140, 50% in each group) mother-infant dyads was estimated based on an 80% statistical power at a 2-tailed α of .05, with the breastfeeding rates of intervention and control groups estimated as 45% and 30%, respectively. In total, 30% was approximately the rate of EBF in China reported by a national survey [6]. Given that a smaller sample size was deemed sufficient and the study duration was extended due to the COVID-19 pandemic, the sample size was re-estimated based on the data from the pilot study.

#### Recruiting Sites

Recruiting sites were reduced from the initial 3 hospitals to 2 sites, as the excluded hospital had a limited number of mothers who met the recruitment criteria.

### Hypotheses

According to Bandura’s theory of social cognition reviewed by Scott et al [29], self-efficacy can support breastfeeding, and performance accomplishments affect self-efficacy [23]. Therefore, the more information a mother knows about her baby’s feeding and nutritional status, the less anxiety she has and the more confident she feels. The study hypotheses were as follows: (1) mothers who used the app to monitor breastfeeding practice, including breastfeeding time and frequency, and to obtain feedback on breast milk and nutrients intake, and who compared monitoring data with recommendations, were more likely than those who used only WeChat to maintain breastfeeding practices; and (2) the more frequently mothers used the app, and the more their self-awareness of breastfeeding increased through assessments and feedback provided by the app, the more likely they were to maintain breastfeeding practices.

### Description of the Intervention and Control Groups

**Figure 1:**
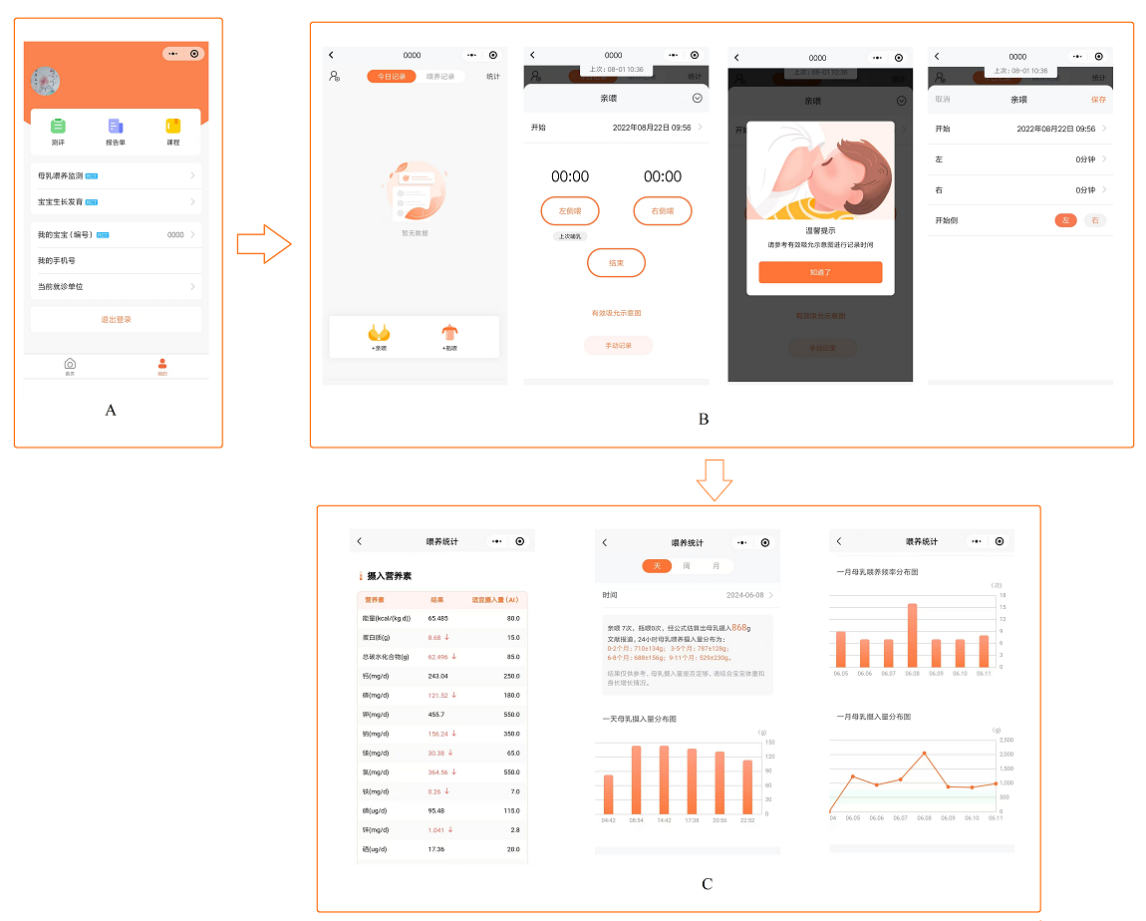
Profile of Breastfeeding Aiding Tool: (A) home page, (B) monitoring pages, and (C) feedback pages.

The app was developed based on our previous study, which explored the factors affecting the breast milk intake of one feed [30], and it is compatible with both Android and iOS devices. A health promotion software developer, 2 childcare experts, 2 medical informaticists, and end users were involved in creating the app. The design of the app page was user-friendly. It included icons with a small amount of text, and there was a link on the home page to evaluate the infant’s developmental quotient. As shown in the app profiles (Figure 1), the monitoring component of the app was designed to collect total breastfeeding time and frequency over a 24-hour period. There were 2 ways for mothers to record breastfeeding time: (1) the app recorded automatically based on when the mother clicked the start and end buttons for each breastfeeding event; (2) mothers timed themselves and entered the breastfeeding time manually. Mothers were shown a photograph of a sucking baby before they started to record breastfeeding time to ensure that they recorded effective breastfeeding time. The feedback section provided tailored information on breast milk, nutrient intake, and the pattern of breastfeeding over time. Nutrients were calculated according to the breast milk composition of Chinese mothers [31]. Recommendations for daily breastfeeding frequency, daily breast milk intake, and nutrient intake were displayed as references for mothers to judge their own breastfeeding status [32,33].

The app (version 1.0) was downloaded and installed on the mothers’ phones after they scanned a QR code using WeChat. The QR code was printed on a booklet (Multimedia Appendix 1) delivered to mothers, and the digital QR code was sent to mothers via WeChat as a reminder. All mothers used the app for free. The study assumed that the participants could fully understand how to use the app and would record their breastfeeding practices truthfully. App use was recorded automatically once the mothers in the intervention group started to track breastfeeding. If they did not use the app, there was no record of use. Mothers were encouraged to use the app for a 2-month period. The scheduled use time was 14 days: 5 days for the first week, 2 days in each of the second and third weeks, and 1 day in each of the maintenance weeks (Multimedia Appendix 1).

Mothers in the control group only had access to the WeChat group and were also followed up for 2 months. The two WeChat groups were created, and a QR code was generated for each group. Mothers were requested to use their WeChat app to scan the QR code to enable them to join their respective groups.

Three health care workers, who were part of the research team, participated in both the intervention and control WeChat groups. For each group, the health care workers (1) followed up with the mothers and sent the digital assessment package; (2) sent digital breastfeeding educational materials once a week; and (3) provided non–face-to-face breastfeeding counseling services to mothers, including breastfeeding advice during the COVID-19 pandemic.

### Data Collection

Once mothers had consented, researchers administered a baseline survey to all participants that included demographic details, maternal and child health status, feeding information, self-reported breastfeeding self-efficacy, and depression status measurements. Data were also collected at the 1-month and 2-month follow-up time points. During each pre- and postintervention appointment, all mothers completed the follow-up digital questionnaire pack consisting of feeding practice, maternal breastfeeding self-efficacy, and depression evaluation. Only feeding practices were collected at 1-month follow-up. All the data were self-reported by participants and collected via a online survey platform SoJump (Changsha Ranxing Information and Technology Co. Ltd), which is widely used in China [34,35]. Data obtained through the use of the app for breastfeeding surveillance consisted of the output from the platform.

Quality assurance methods were as follows: (1) a detailed operation manual was provided for operators to follow; (2) a teaching video (Multimedia Appendix 2) explaining how to use the app was sent to the intervention WeChat group to help mothers enter accurate information; and (3) researchers imported data from the platform at least every 2 weeks to check for data with errors and omissions, and promptly communicated with participants via WeChat and phone calls to correct and supplement the data.

Measures included a sociodemographic questionnaire, including infants’ age and sex, mothers’ age, education, and occupation; feeding practices, which were self-reported in the past 1 month, and included average breastfeeding frequency per day, average formula feeding frequency per day, and complementary feeding information; the Breastfeeding Self-Efficacy Scale–Short Form, which was developed by Dennis et al [36] to assess maternal breastfeeding self-efficacy and used globally among numerous maternal populations (it consists of 14 self-reported items scored from 1 [not at all confident] to 5 [always confident]; higher scores indicated greater self-efficacy of breastfeeding; the α coefficient of the Chinese version was 0.927 [37]) and the Center for Epidemiological Survey–Depression Scale (CES-D), which consists of 20 self-reported items and has been used extensively to screen for postnatal depression. Items were scored 0, 1, 2, or 3, depending on the frequency of symptoms (rarely, sometimes, occasionally, or all the time). Depression can be adequately measured and screened for using the single-factor structure underlying the CES-D scores in China [38,39].

At the end of the study, mothers in the intervention group were invited to participate in interviews conducted via WeChat to obtain qualitative feedback. Nonusers were asked for their reasons for not using the app, and users were asked to evaluate their satisfaction with using the app. The questions in the satisfaction interview referred to the app’s functions, esthetics, information, and interaction, and were adapted from the user version of the Mobile Application Rating Scale, which is used to assess the quality of mHealth apps [40].

### Outcome Indicators

#### Primary Outcomes

On the basis of the WHO definition, the patterns of breastfeeding were defined as (1) EBF, where infants received only breast milk; (2) predominant breastfeeding (PBF), where infants received breast milk as the predominant source of nourishment, along with water or water-based drinks, fruit juice, and water-based calcium supplements in this study; and (3) partial breastfeeding, which refers to mixed feeding (MF) of breast milk and other food or food-based fluids, such as formula milk or weaning foods [35,41]. For the main primary outcomes, we combined EBF and PBF into a single variable, as full breastfeeding (FBF). Therefore, the pattern of breastfeeding as a categorical variable was defined as FBF or MF.

Maternal breastfeeding self-efficacy: is another primary outcome, which was assessed using the Breastfeeding Self-Efficacy Scale–Short Form score.

#### Secondary Outcomes

The secondary outcomes were breastfeeding frequency, which is the number of breastfeeding sessions per day, and maternal depression status, assessed using the CES-D score. Considering that postpartum depression affects approximately 23.5% of the population in China [42] and is an important aspect of maternal psychological status, it was negatively related to EBF and breastfeeding self-efficacy, as reported in some studies [43,44].

### Statistical Analysis

Missing values for the feeding practices data collected at the 1-month follow-up time point were randomly imputed using the values from the baseline and 2-month follow-up time points.

The data were analyzed using the SPSS statistical software (version 20.0 for Windows; IBM Corp). Descriptive statistics were calculated for all variables of interest. Numerical variables were presented as means and SDs to describe the distribution, and categorical variables were presented as proportions.

Two statistical approaches were used: (1) intention-to-treat (ITT; intervention vs control) approach for primary analysis and (2) per protocol (PP; using the tool vs not using the tool) approach for secondary analysis. PP analysis aimed to confirm the intervention effects, and protocol adherence was derived from the app data. However, the sample became self-selected rather than an unbiased sample from a randomized trial. Furthermore, mothers who did not adhere to the random assignment were included. As a result, the benefits of randomization, such as the elimination of systematic errors, were lost [45-47].

Tool use was considered positive when there were any use records from mothers, regardless of the number of records; even a single record was considered use. The 2-tailed *t* test and chi-square or Fisher exact test were used to investigate the differences between the two groups in ITT and PP analyses.

Bivariate logistic regression was used to obtain the odds ratio (OR) and adjusted OR (aOR). Enter regression was implemented. EBF, PBF, FBF, and MF were the dependent variables; intervention group in the ITT sample and tool use group in the PP sample were the independent variables, respectively. ORs and aORs were presented along with their associated 95% CIs. aORs were adjusted for 11 variables: maternal education level, maternal job status, maternal age, diseases during pregnancy, maternal BMI, breastfeeding initial time, infant birth weight, gestational age, infant sex, child illnesses, and breast problems. The selection of independent variables to be incorporated into the model was based on a specific statistical criterion. In the single-factor chi-square tests conducted for each variable and for EBF, PBF, FBF, or MF, only those variables with *P* values <.3 were considered for inclusion. This approach aimed to incorporate as many individual factors on breastfeeding as possible to provide a comprehensive analysis [11].

The changes between baseline and postintervention time point in each group were compared to show the effectiveness of the intervention more clearly. The criterion for significance was set at α=.05.

### Ethical Considerations

The Capital Institute of Pediatrics Ethics Committee (SHERLL2022033) approved the study according to the International Organizations of Medical Sciences on human biomedical research international guidelines and ethical principles from the Declaration of Helsinki.

The purpose and content of the study were explained to the participants, and informed consent was obtained in the clinics before they entered their groups. To obtain consent, mothers were asked to read through and sign the consent forms (Multimedia Appendix 3), and researchers provided explanations if participants could not understand any part of the consent form. All the participants were told that information about their demographic details, maternal and child health status, feeding information, self-reported breastfeeding self-efficacy, and depression status would be collected.

The research process followed the privacy and confidentiality protocol. All data were archived on a project-dedicated computer and maintained by project-dedicated personnel. To ensure anonymity, before recording the data, participants were assigned a numeric identifier, rather than using their and their children’s names.

All participants in both groups were provided with educational and consultation services. In addition, each mother who completed the follow-up received a parenting guidebook.

## Results

### Recruitment and Dropout Analysis

Recruitment started in September 2022 during the COVID-19 pandemic and continued until October 2023; the intervention period lasted until January 2024. The follow-up was not affected by the pandemic, but the recruitment in the clinics lasted longer than anticipated.

**Figure 2:**
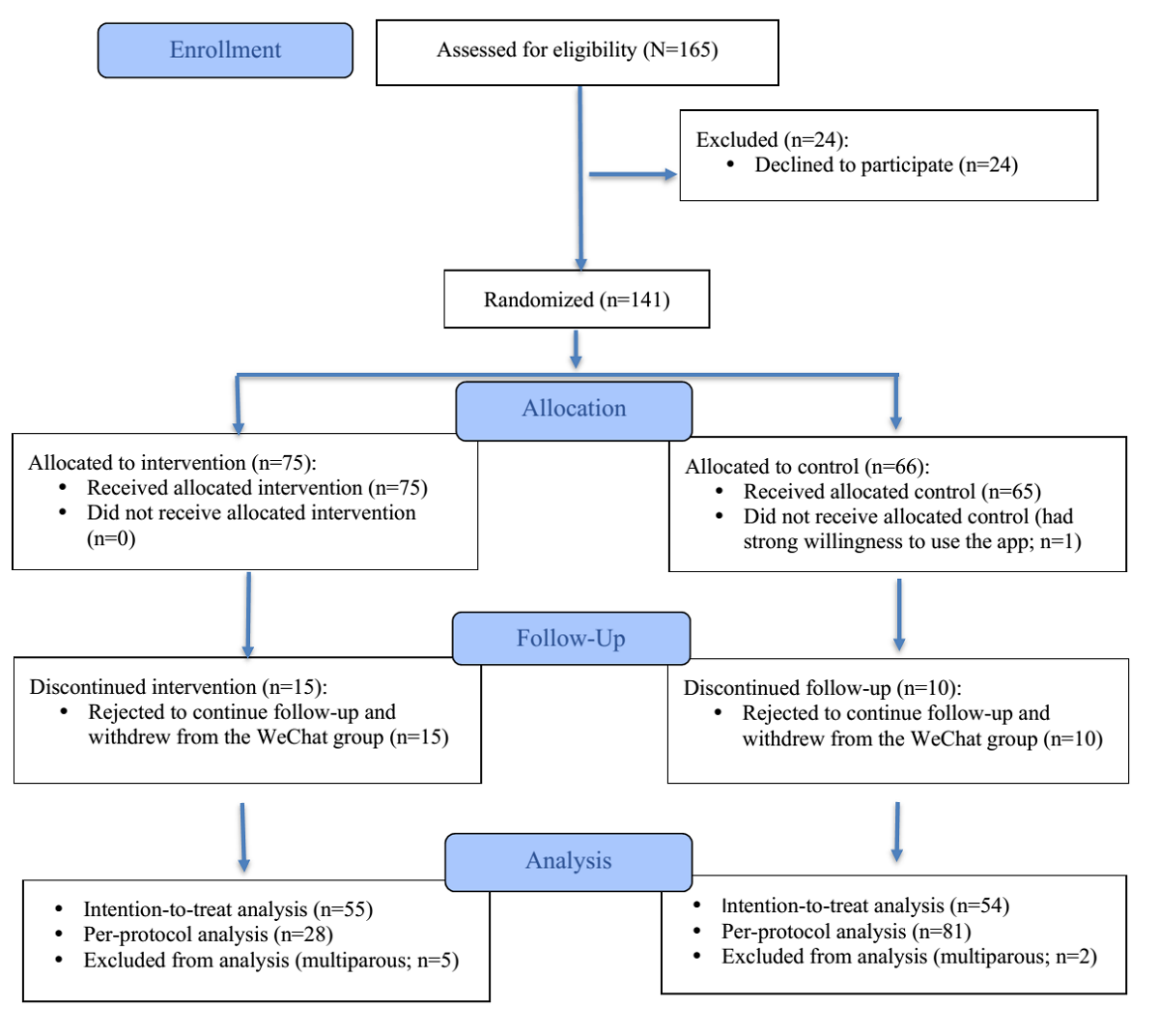
CONSORT (Consolidated Standards of Reporting Trials) flow diagram.

Figure 2 illustrates recruitment, follow-ups, and dropouts, based on the CONSORT-EHEALTH (Consolidated Standards of Reporting Trials of Electronic and Mobile Health Applications and Online Telehealth) criteria [45]. A total of 141 eligible and consenting mother-infant dyads (n=75, 53.2% in intervention group vs n=66, 46.8% in the control group) were allocated to the two groups. Of these, 25 mothers dropped out (n=15, 60% in intervention group vs n=10, 40% in control group) because they rejected follow-ups or withdrew from the WeChat groups; 7 mothers (n=5, 71% in the intervention group vs n=2, 29% in the control group) were excluded from analysis for multiparity. The attrition rate was 17.7% (25/141) in total, with 20% (15/75) in the intervention group and 15% (10/66) in the control group. Overall, 109 mothers (n=55, 50.5% in the intervention group vs n=54, 49.5% in the control group) were included in the final sample for analysis. One mother who was initially allocated to the control group preferred to use the app; therefore, she was counted in the control group in the ITT sample and the tool use group in the PP sample. No adverse events were recorded or reported.

### Characteristics of Participants

Table 1 presents the baseline characteristics of the participants. Mothers had a mean age of 31.36 (SD 3.38) years. In total, 93.6% (102/109) of the mothers had a college-level education or above. In the intervention group, 49% (27/55) of the mothers had records of using the app. In total, 28 mothers used the app, of whom 14 (50%) mothers had records of 1 to 13 days (2 mothers had a single record), and 14 (50%) mothers had records of >14 days. The average length of use was 12 days.

**Table 1:** Baseline characteristics of participants (N=109).

Characteristics		ITT^a^ intervention (n=55)	ITT control (n=54)	*P* value		PP^b^ group using the tool (n=28)		PP group not using the tool (n=81)		*P* value	
Maternal age (y), mean (SD)		32.04 (3.32)	30.66 (3.34)	*.04^c^*		32.32 (2.92)		31.01 (3.49)		.08	
**Maternal education level, n (%)**					.29^d^						*.04^c,d^*
	High school or below	3 (5)	1 (2)			0 (0)		4 (5)			
	College	28 (51)	34 (63)			12 (43)		50 (62)			
	Master or above	23 (42)	17 (31)			16 (57)		24 (30)			
C-section, n (%)		13 (24)	14 (26)	.78		4 (14)		23 (28)		.14	
Sex, male, n (%)		24 (44)	28 (52)	.39		11 (39)		41 (51)		.30	
Gestational age at birth (wks), mean (SD)		39.51 (1.03)	39.48 (0.92)	.88		39.64 (1.10)		39.44 (0.93)		.73	
Birthweight (g), mean (SD)		3305.64 (334.78)	3357.59 (348.23)	.43		3342.86 (320.76)		3327.41 (349.47)		.84	
Maternal BMI (kg/m^2^), mean (SD)		22.98 (2.43)	23.77 (3.34)	.16		23.01 (2.48)		23.50 (3.08)		.45	
Diabetes during pregnancy, n (%)		16 (29)	13 (24)	.55		9 (32)		20 (25)		.44	
Hypertension during pregnancy, n (%)		3 (5)	3 (6)	>.99^a^		0 (0)		6 (7)		.34 ^d^	
Anemia during pregnancy, n (%)		17 (31)	11 (20)	.21		8 (29)		20 (25)		.68	
**Maternal job status, n (%)**					.69						.50
	Housewife/full maternity leave	41 (74)	42 (78)			20 (71)		63 (78)			
	Maternity leave and working	14 (25)	12 (22)			8 (29)		18 (22)			
Alcohol, n (%)		1 (2)	0 (0)	>.99^d^		1 (4)		0 (0)		.26^d^	
**Breastfeeding initial time, n (%)**					.83						.92
	Less than 1 h	20 (36)	22 (41)			10 (36)		32 (40)			
	1 h-1 day	18 (33)	18 (33)			10 (36)		26 (32)			
	1 day later	17 (31)	14 (26)			8 (29)		23 (28)			
Reported breast problems, n (%)		18 (33)	10 (18)	.09		12 (43)		16 (20)		*.02^c^*	
Reported insufficient breastmilk, n (%)		9 (16)	13 (24)	.32		6 (21)		16 (20)		.85	
Reported child with illness, n (%)		26 (47)	20 (37)	.28		14 (50)		32 (40)		.33	

^a^ITT: intention-to-treat.

^b^PP: per protocol.

^c^Significance of values in italics: maternal age in ITT (*P*=.04), maternal education level in PP (*P*=.04), and reported breast problems in PP (*P*=.02).

^d^Fisher exact test.

Baseline characteristics were balanced between conditions for ITT and PP samples, except for maternal age in the ITT sample (*P*=.04). Mothers using the tool had greater maternal education (high proportion of mothers with masters degree or above, *P*=.04) and had more breast problems (nipple pain and mastitis) in the PP sample (*P*=.02). There was no significant difference in reported sufficiency of breast milk between the 2 groups of mothers in the ITT and PP samples (*P*=.32 and *P*=.85 respectively). No mother smoked, and no infant had neonatal asphyxia. The attrition analysis (Multimedia Appendix 4) showed that it was balanced between mothers who completed the follow-up and those who dropped out, except for the sex of the infants (*P*=.04; dropouts among mothers with male children: 6/58, 10%; dropouts among mothers with female children: 19/25, 76%). More details see Multimedia Appendix 4.

### Impact of the Mobile App on Breastfeeding Practices

At baseline, the rates of EBF, PBF, FBF, and MF were similar (all *P*>.05 in the ITT and PP samples), as shown in Table 2.

**Table 2:** Breastfeeding practices at different time points (N=109).

Time points			ITT^a^ intervention group (n=55)		ITT, control group (n=54)		Chi-square (*df*)		*P* value		OR^b^ (95% CI)		aOR^c^ (95% CI)		PP^d^ group using the tool (n=28)		PP group not using the tool (n=81)		Chi-square (*df*)		*P* value		OR (95% CI)		aOR (95% CI)
**Baseline, n (%)**																									
	EBF^e^ rate	25 (45)		27 (50)		0.2 (1)		.64		0.83 (0.39-1.77)		0.96 (0.38-2.42)		12 (43)		40 (49)		0.4 (1)		.55		0.77 (0.32-1.83)		0.66 (0.23-1.90)	
	PBF^f^ rate	16 (29)		13 (24)		0.4 (1)		.55		1.29 (0.55-3.04)		1.72 (0.63-4.66)		10 (36)		19 (23)		1.6 (1)		.21		1.81 (0.72-4.59)		2.87 (0.91-9.08)	
	FBF^g^ rate	41 (74)		40 (74)		0.003 (1)		.96		1.02 (0.43-2.42)		1.68 (0.59-4.79)		22 (79)		59 (73)		0.4 (1)		.55		1.37 (0.49-3.82)		1.73 (0.53-5.69)	
	MF^h^ rate	14 (25)		14 (26)		0.003 (1)		.96		0.98 (0.41-2.30)		0.60 (0.21-1.70)		6 (21)		22 (27)		0.4 (1)		.55		0.73 (0.26-2.04)		0.58 (0.18-1.91)	
**1-month follow-up, n (%)**																									
	EBF rate)	30 (54)		33 (61)		0.5 (1)		.49		0.76 (0.36-1.64)		1.20 (0.47- 3.08)		14 (50)		49 (60)		0.9 (1)		.33		0.65 (0.28-1.55)		0.83 (0.29-2.39)	
	PBF rate	15 (27)		8 (15)		2.5 (1)		.11		2.16 (0.83-5.62)		2.83 (0.77-10.35)		10 (36)		13 (16)		*4.8* (1)		*.03*		2.91 (1.10-7.70)^i^		4.81 (1.12-20.78)^i^	
	FBF rate	45 (82)		41 (76)		0.6 (1)		.45		1.43 (0.56-3.60)		3.31 (0.98-11.16)		24 (86)		62 (76)		1.05 (1)		.30		1.84 (0.57-5.96)		2.99 (0.74-12.15)	
	MF rate	10 (18)		13 (24)		0.6 (1)		.45		0.70 (0.28-1.77)		0.30 (0.09-1.02)		4 (14)		19 (23)		1.05 (1)		.30		0.55 (0.17-1.76)		0.33 (0.08-1.36)	
**2-month follow-up, n (%)**																									
	EBF rate	29 (53)		26 (48)		0.2 (1)		.63		1.20 (0.57-2.55)		1.75 (0.71-4.34)		16 (57)		39 (48)		0.7 (1)		.41		1.44 (0.60-3.41)		1.66 (0.59-4. 68)	
	PBF rate	17 (31)		13 (24)		0.6 (1)		.42		1.41 (0.60-3.29)		1.43 (0.52-3.96)		10 (36)		20 (25)		1.3 (1)		.26		1.69 (0.67-4.27)		1.84 (0.57-5.95)	
	FBF rate	46 (84)		39 (72)		2.1 (1)		.15		1.97 (0.78-4.98)		3.16 (1.05-9.54)^i^		26 (93)		59 (73)		*4.9*^j^ (1)		*.03*		4.85 (1.06-22.15)^i^		5.55 (1.07-28.83)^i^	
	MF rate	9 (16)		15 (28)		2.1 (1)		.15		0.51 (0.20-1.29)		0.32 (0.10-0.95)^i^		2 (7)		22 (27)		*4.9* (1)		*.03*		0.21 (0.04-0.94)^i^		0.18 (0.4-0.94)^i^	

^a^ITT: intention-to-treat.

^b^OR: odds ratio.

^c^aOR: adjusted odds ratio.

^d^PP: per protocol.

^e^EBF: exclusive breastfeeding.

^f^PBF: predominant breastfeeding.

^g^FBF: full breastfeeding.

^h^MF: mixed feeding.

^i^*P*<.05.

^j^Significance of values in italics: PBF rate in PP (*P*=.03) at 1 month time point, FBF rate (*P*=.03), and MF rate (*P*=.03) in PP at the 2-month time point.

After the 2-month follow-up, the EBF rate of the tool-using group (tool and WeChat) was 57% (16/28), compared to 48% (39/81) in the group not using the tool (WeChat only). The parameters for the PP sample were *P*=.41; OR 1.44, 95% CI 0.60-3.41; and aOR 1.66, 95% CI 0.59-4.68. The FBF rate was significantly higher in the mothers in the tool-using group than in those not using the tool (26/28, 93% vs 59/81, 73%. The parameters for the PP sample were *P*=.03; OR 4.85, 95% CI 1.06-22.15; and aOR 5.55, 95% CI 1.07-28.83. The aOR (3.16, 95% CI 1.05-9.54) of the intervention and control groups in the ITT sample was significant. After the 2-month follow-up, the significant results for the MF rate were similar to those for the FBF rate. In the PP sample, after the 1-month follow-up, the PBF rate was significantly higher in the group of mothers using the tool than in the group not using the tool (10/28, 36% vs 13/81, 16%; *P*=.03; OR 2.91, 95% CI 1.10-7.70; and aOR 4.81, 95% CI 1.12-20.78). There were no significant differences in the EBF rate between the 2 groups in the ITT and PP samples after the 1-month and 2-month follow-up (1-month: *P*=.49 and *P*=.33; 2-month: *P*=.63 and *P*=.41). There were also no significant differences in the FBF rate between the 2 groups in the ITT sample after the 1-month and 2-month follow-up (*P*=.45 and *P*=.15).

As shown in Table 3, there were no significant differences in the postintervention-preintervention changes of breastfeeding frequency between the 2 groups in the ITT and PP samples (*P*=.96 and *P*=.60).

**Table 3:** Breastfeeding self-efficacy and frequency at different time points.

Time points			ITT^a^ intervention group (n=55)		ITT control group (n=54)		*t* test (*df*)		*P* value		PP^b^ group using the tool (n=28)		PP group not using the tool (n=81)		*t* test (*df*)		*P* value
**Baseline, mean (95% CI)**																	
	BSES-SF^c^ score	51.78 (48.35 to 55.21)		51.59 (47.24 to 55.94)		0.07 (107)		.94		50.93 (46.59 to 55.26)		51.95 (48.57 to 55.34)		0.32 (107)		.75	
	CES-D^d^ score	27.47 (25.09 to 29.85)		25.83 (23.97 to 27.70)		1.08 (107)		.28		29.89 (25.74 to 34.04)		25.54 (24.13 to 26.95)		*2.03* *(34)* ^e^		*.05*	
	24 hour breastfeeding frequency	9.19 (8.72 to 9.66)		8.80 (8.15 to 9.45)		0.99 (107)		.33		9.09 (8.30 to 9.88)		8.96 (8.50 to 9.43)		0.28 (107)		.78	
**2-month follow-up, mean (95% CI)**																	
	BSES-SF score	51.91 (48.00 to 55.82)		51.93 (47.29 to 56.56)		0.01 (107)		1.00		52.29 (47.12 to 57.45)		51.79 (48.14 to 55.44)		0.14 (107)		.89	
	CES-D score	27.25 (25.00 to 29.51)		26.48 (23.90 to 29.07)		0.45 (107)		.65		27.61 (24.06 to 31.15)		26.62 (24.66 to 28.57)		0.50 (107)		.61	
	24 h breastfeeding frequency	7.52 (7.18 to 7.85)		7.15 (6.54 to 7.75)		1.07 (83)^e^		.29		7.62 (7.07 to 8.18)		7.24 (6.81 to 7.66)		0.99 (107)		.32	
**Postintervention-preintervention changes, mean (95% CI)**																	
	BSES-SF score	0.13 (–3.69 to 3.94)		0.33 (–3.18 to 3.85)		0.08 (107)		.94		1.36 (–3.79 to 1.50)		–0.16 (–3.16 to 2.84)		0.51 (107)		.61	
	CES-D score	–0.22 (–2.25 to 1.82)		0.65 (–1.46 to 2.76)		0.59 (107)		.55		–2.29 (–5.19 to 0.62)		1.07 (–0.58 to 2.73)		*2.05* *(107)* ^f^		**.** *046*	
	24-hour breastfeeding frequency	–1.67 (–2.14 to –1.20)		–1.65 (–2.41 to –0.89)		0.06 (107)		.96		–1.46 (–2.18 to –0.75)		−1.73 (–2.27 to –1.19)		0.52 (107)		.60	

^a^ITT: intention-to-treat.

^b^PP: per protocol.

^c^BSES-SF: breastfeeding Self-Efficacy Scale–Short Form.

^d^CES-D: Center for Epidemiological Survey–Depression Scale.

^e^Unequal variances.

^f^Significance of values in italics: CES-D score in PP at 1-month time point (*P*=.05) and between postintervention and preintervention (*P*=.046).

**Figure 3:**
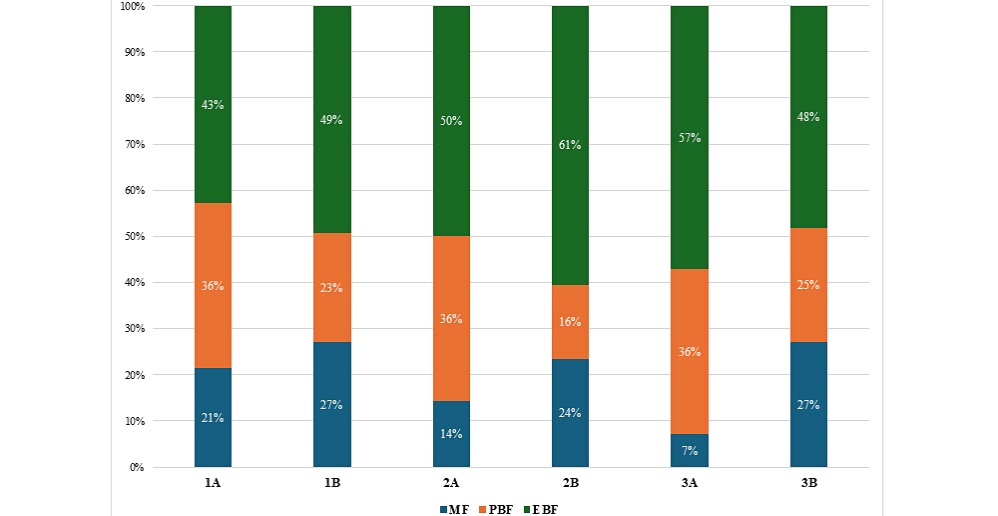
Proportions of exclusive breastfeeding (EBF), predominant breastfeeding (PBF), and mixed feeding (MF) between the groups using and not using the tool. 1A. the group using the tool at baseline; 1B: the group not using the tool at baseline; 2A: the group using the tool at 1-month follow-up; 2B: the group not using the tool at 1-month follow-up; 3A: the group using the tool at 2-month follow-up; 3B: the group not using the tool at 2-month follow-up.

As shown in Figure 3, after the intervention, the EBF rate in the group that used the tool was 57% (16/28), while it was only 48% (39/81) in the group that did not use the tool (*P*=.41); the PBF rate was 36% (10/28) in the tool-using group and 25% (20/81) in the non–tool-using group (*P*=.26); as well as the MF rate was 7% (2/28) in the group using the tool and 27% (22/81) in the group that did not use the tool (*P*=.03). There was a decreasing shift of the MF rate among the app users from 21% (6/28) to 7% (2/28) over a 2-month intervention period. A total of 4 mothers ceased using formula altogether, while the others’ breastfeeding patterns remained the same. Feeding tracking data revealed that 3 (75%) mothers transitioned to PBF, while 1 (25%) mother transitioned to EBF.

### Impact of the Mobile App on Maternal Breastfeeding Self-Efficacy

As shown in Table 3, the mothers’ breastfeeding self-efficacy scores were similar in the ITT and PP samples at baseline and 2-month follow-up. The mothers’ breastfeeding self-efficacy was enhanced by approximately 1.36 on average (95% CI –3.79 to 1.50) in the group using the tool and decreased by 0.16 (95% CI –3.16 to 2.84) in the group not using the tool; the difference was not significant (*P*=.61).

At both baseline and 2-month follow-up, the depression scores of the mothers were similar for the groups in the ITT sample. The score was marginally significantly higher in the group using the tool (29.89, 95% CI 25.74-34.04 vs 25.54, 95% CI 24.13-26.95; *P*=.05) at baseline. The mothers’ depression score decreased by 2.29 on average (95% CI –5.19 to 0.62) in the group using the tool, while it increased by 1.07 (95% CI –0.58 to 2.73) in the group not using the tool; the change was significant (*P*=.046).

### Results of Qualitative Feedback

In total, 7 mothers provided qualitative feedback, of whom 3 (43%) mothers who used the app provided positive evaluations in some of its functions, esthetics, and information; however, they considered that the app lacked customized settings and prompts. The remaining 4 (57%) mothers did not want to use the app due to personal preference, for example, some preferred paper-based records or other feeding apps, and some thought it was troublesome to record breastfeeding and forgot to use the app (Multimedia Appendix 5).

## Discussion

### Principal Findings

This study represents the first randomized controlled trial investigating an app-based intervention comprising 2 core elements: non–face-to-face monitoring of breastfeeding time and personalized feedback tailored to primiparous mothers in urban China. The objective was to evaluate its impact on EBF and maternal self-efficacy pertaining to breastfeeding as compared to the control condition, which was an education and consultation program via WeChat. Notably, the ITT analysis failed to reveal a statistically significant intervention effect, primarily attributed to the fact that approximately half (28/55, 51%) of the mothers did not use the app-based program. The PP results demonstrated significant improvements in breastfeeding and a decrease in the use of formula at 2 months in the mothers who actively used the app compared to those who did not at 2 months. Although this study did not show significance in the effectiveness of the app in increasing EBF, it did show the app’s ability to improve breastfeeding practices and reduce formula intake. Previous studies using interventions such as a breastfeeding diary to monitor lactation also showed that monitoring was an effective way to improve breastfeeding [24,48]. Dinour [24] reported that compared with nonusers, mothers who used apps to track breastfeeding were more likely to have ever breastfed and exclusively breastfed their infants. Nevertheless, some mHealth intervention studies also encountered difficulties in improving EBF or promoting longer EBF periods. For example, the study by Bunik et al [28] showed no evidence of difference between the intervention and the control groups with regard to EBF, and the study by Wu et al [35] showed no significance in EBF during the past 2-month follow-up.

There were several potential reasons for our study’s failure to have a significant effect on EBF. First, in the definition of EBF, infants should not be provided with water; however, mothers in our study gave water to babies mainly for nutritional supplementation, in particular calcium. Pediatricians in China suggest calcium supplementation for infants’ bone mineral density [49], especially in the developed areas; this may have been a more critical problem for EBF promotion than formula use. The study by Ahmed et al [22] proved the effectiveness of the intervention of an online, interactive breastfeeding monitoring system on EBF, with a low rate of PBF in the whole sample. At the same time, it revealed that the provision of water to locally breastfeeding infants was an uncommon practice, potentially stemming from higher maternal anxiety about nutritional well-being among women in the Chinese sample versus those from other countries. This divergence could also be attributed to a different understanding of the WHO’s concept of EBF, which emphasizes the sole reliance on breast milk for nourishment and no water provision during the initial months of life [50].

It is noteworthy that the group that actively used the tool had a higher EBF rate compared to the group that did not use it. However, the small sample size in the app-engaged group hindered the study’s ability to achieve statistical significance.

According to the results, mothers’ self-efficacy in the group using the tool was enhanced by approximately 1.36, and that of the group not using the tool decreased by approximately 0.16. The mothers’ depression score in the group using the tool decreased by approximately 2.29, and that of the group not using the tool increased by 1.07, resulting in a significant difference. The results suggest that using the app may have a positive impact on breastfeeding mothers’ emotional well-being. This finding is supported by Qian et al [17], whose review showed that mHealth interventions could improve maternal well-being and reduce anxiety. Furthermore, Ahmed and Roumani [23] and Dienelt et al [26] proved that a breastfeeding monitoring app or infant feeding apps with a feeding tracker component provided mothers with a perception of greater control, confidence, and efficacy about infant feeding. The study by Ahmed and Roumani [23] differed from this study in including tailored feedback via notifications for breastfeeding problems, such as the inability to latch or insufficient feeding. This suggests that by addressing a diverse range of their needs, mothers’ confidence in breastfeeding can be effectively enhanced.

In this study, all the mothers were recruited from 2 hospitals in Beijing and had higher educational levels, with 93.6% (102/109) holding a college degree or above, and among mothers who use the app, a high proportion held a masters degree or above. They demonstrated a high-level ability to use the internet to improve their breastfeeding skills, which may limit the study’s generalizability to the general population.

In addition, the data show that mothers who used the app had significantly more breast problems at baseline and a higher depression score than mothers who did not use it. This suggests that mothers with more breast problems and postpartum depression symptoms might want to seek additional help and were more willing to accept and use the intervention. Previous studies showed that mothers with postpartum depression may have a higher risk of non EBF without additional support and consultation [43,51]. In our study, mothers with initial higher depression scores who used the app were more willing to maintain breastfeeding, and final depression scores tended to be lower. This corresponds with the study by Henshaw [52], which showed that women who breastfed exclusively had a lower likelihood of developing significant postpartum depression.

According to the qualitative feedback, the app’s simplicity and targeted functionality were well-received by some (3/7, 43%) mothers who found that it provided the information and features they required. We believe that the app will enable health care workers to counsel mothers to sustain breastfeeding via WeChat group online or even in clinics offline. Approximately half (27/55, 49%) of the mothers offered the opportunity to use a new monitoring app did not choose to use it. The most likely explanations for nonuse are that some mothers found it inconvenient to use, or it may not have been sufficiently multifunctional to appeal to others. To further improve the app’s accessibility and acceptability for new mothers, we propose the following strategies:

- Develop customized settings and prompts within the app to enhance its personalization. This will enable mothers to tailor the app’s content and notifications to their specific needs and preferences, thereby increasing their engagement and satisfaction.
- Consider integrating the app with wearable devices to provide mothers with greater convenience. This will allow for seamless data tracking and real-time feedback, making it easier for mothers to monitor their breastfeeding progress and receive timely support.
- Testing with women of different education levels to ensure its usability and effectiveness across a broader demographic.

### Methodological Limitations

Methodological limitations must be acknowledged in this eHealth intervention study. The inherent and potential risk of measurement bias due to unblinding was an unavoidable factor [53]. Specifically, one mother in the control group used the app, which introduced bias into the intention-to-treat sample analysis. All questionnaires and assessments were self-reported, and this relied solely on individual integrity, which could induce potential bias. Self-reporting can be influenced by various factors, such as recall errors, which may affect the accuracy and reliability of the data [54].

### Conclusions

This study marks the first comparative trial examining the efficacy of non–face-to-face monitoring interventions and feedback mechanisms specifically tailored for primiparous mothers as an aid for breastfeeding guidance in urban China. Leveraging a dedicated mobile app as a supportive tool, our findings indicate a significant trend among participants: a substantial upholding of breastfeeding rates coupled with a reduction in formula supplementation and subsequent improved maternal emotion status associated with the breastfeeding experience. To consolidate these promising results, we urge for further solutions to address the barriers that hinder the acceptance of the existing app, followed by a more extensive trial that incorporates a larger cohort of mothers embracing the use of this app.

## Appendix

Multimedia Appendix 1 Booklet for app use.

Multimedia Appendix 2 Teaching video for the app.

Multimedia Appendix 3 Informed consent form.

Multimedia Appendix 4 Baseline characteristics of participants.

Multimedia Appendix 5 Qualitative feedback information from the participants.

Multimedia Appendix 6 CONSORT-eHEALTH checklist (V 1.6.1).

## Abbreviations

aOR: adjusted odds ratio
CES-D: Center for Epidemiological Survey–Depression Scale
CONSORT-EHEALTH: Consolidated Standards of Reporting Trials of Electronic and Mobile Health Applications and Online Telehealth
EBF: exclusive breastfeeding
FBF: full breastfeeding
ITT: intention-to-treat
MF: mixed feeding
mHealth: mobile health
OR: odds ratio
PBF: predominant breastfeeding
PP: per protocol
WHO: World Health Organization
