# Supplementary figures and images for: Effectiveness of a Mobile Breastfeeding Monitoring Tool Among Mothers in WeChat Groups on Breastfeeding Exclusivity and Self-Efficacy: Intention-to-Treat and Per-Protocol Analyses of a Randomized Controlled Trial
Source: J Med Internet Res. 2025 Aug 15;27:e67024. doi: 10.2196/67024 (PMC12397754; doi:10.2196/67024)

**Multimedia Appendix 1 Booklet**


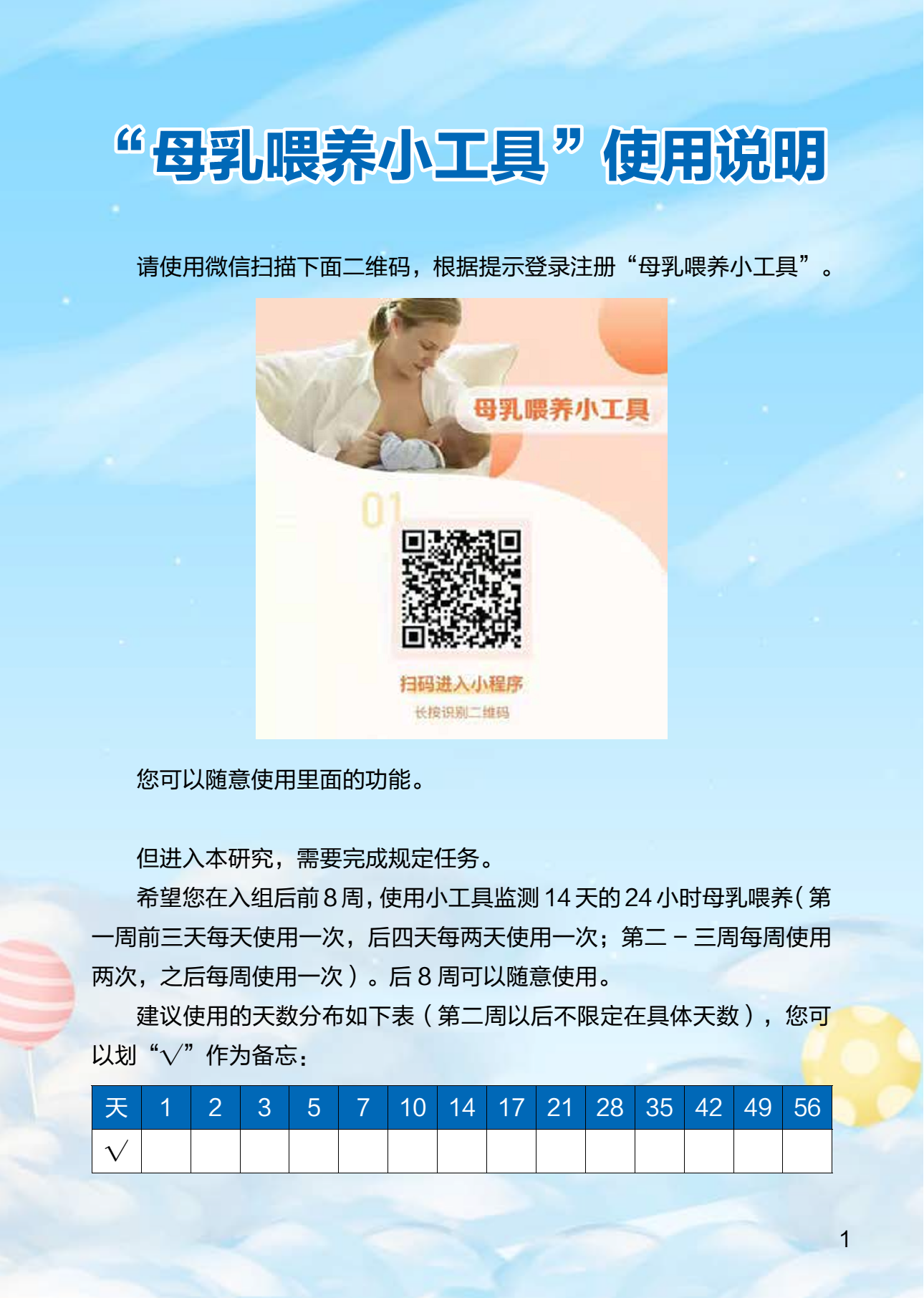

Supplement: Multimedia Appendix 1 [file jmir_v27i1e67024_app1.docx]
