# Supplementary material for: Effectiveness of a Mobile Breastfeeding Monitoring Tool Among Mothers in WeChat Groups on Breastfeeding Exclusivity and Self-Efficacy: Intention-to-Treat and Per-Protocol Analyses of a Randomized Controlled Trial
Source: J Med Internet Res. 2025 Aug 15;27:e67024. doi: 10.2196/67024 (PMC12397754; doi:10.2196/67024)
Supplement: Multimedia Appendix 3 [file jmir_v27i1e67024_app3.docx]

**Multimedia Appendix 3 Informed Consent Form**

Hello, we are going to conduct a study titled “Randomized Controlled Intervention Study on Promoting Breastfeeding through Mobile Healthcare.” As your situation meets the inclusion criteria for this study, we invite you to participate. This informed consent form will introduce the purpose, procedures, benefits, and risks of the study. Please read it carefully before deciding whether to participate. When the researcher explains and discusses the informed consent form with you, you may ask questions at any time and request that he/she explain anything you do not understand. You may also discuss it with your family, friends, and researchers before making a decision.

If you are currently participating in other clinical studies, please be sure to inform your study doctor or researcher.

The principal investigator of this study is Researcher Dai Yaohua from the Capital Institute of Pediatrics. The participating unit study leaders include Chief Physician Yan Qi from the Haidian Maternal and Child Health Hospital and Chief Physician Bao Zheng from the Tongzhou Maternal and Child Health Hospital.

1. **Why is this study being conducted?**

Breastfeeding is crucial for promoting maternal and infant health. For infants, early initiation and exclusive breastfeeding for six months can reduce neonatal infection and mortality rates. Continued breastfeeding for two years or more can also lower the risk of overweight/obesity in children and adolescents. For mothers, longer breastfeeding periods can reduce the risk of breast cancer, ovarian cancer, and type 2 diabetes. Despite the many benefits of breastfeeding, the global breastfeeding rate is low. The latest data in our country shows that the exclusive breastfeeding rate for infants aged 0-6 months is 28%. One of the goals of the State Council's "China Children Development Outline (2021-2030)" is to increase the exclusive breastfeeding rate for infants aged 0-6 months to over 50%.

With the rapid increase in the popularity of mobile devices, mobile healthcare, as a new form of health education, has quickly emerged in the medical field due to its advantages of not being constrained by time and space and saving human and material resources. This study aims to provide breastfeeding guidance based on mobile healthcare to enhance mothers' breastfeeding self-efficacy and promote breastfeeding outcomes.

1. **Who will be invited to participate in this study?**

Inclusion criteria: 1) Primiparous; 2) Healthy infants around 42 days (35-49 days) old (gestational age ≥37 weeks, birth weight ≥2500g, Apgar score ≥9); 3) No major health issues found in the infant's routine physical examination; 4) No congenital or infectious diseases in the infant after screening; 5) No problems with the infant's sucking and swallowing movements; 6) Mother plans to exclusively breastfeed and is in the lactation phase; 7) Mother has no diseases that affect normal breastfeeding.

1. **How many people will participate in this study?**

This study plans to recruit 300 participants, to be conducted at three clinical research centers. There will be 150 participants in the intervention group and 150 in the control group.

1. **How long is the expected duration of participation in the trial?**

The intervention group will receive an 8-week intervention, followed by an 8-week observation and follow-up period, making the total study duration 16 weeks. The control group will participate in surveys and follow-ups for 16 weeks.

1. **How will this study be conducted?**

All information collection in this study will be carried out through WeChat. It is recommended that you set a WeChat password to ensure the security of information entry. You will need to fill in relevant survey forms and assessment scales on time, and researchers will communicate with you by phone about the filling situation and any verification needed.

After signing the informed consent form, you will be assigned a number. You will be randomly assigned (like flipping a coin) to one of the two groups (intervention group and control group). The probability of being assigned to each group is 50%. Both the intervention and control groups will receive breastfeeding consultation and guidance.

What the intervention group needs to do: For the first 8 weeks, use the “Breastfeeding Aiding Tool” for 14 days, input information according to the process, and assess the 24-hour breastfeeding intake (see detailed instructions). At each time point, we will check for any missing information from the backend and provide timely feedback by phone.

Both the intervention and control groups need to fill in questionnaires and self-assessment scales at the follow-up time points. At each follow-up point, we will communicate with you by phone, and you can also choose to visit the outpatient clinic for breastfeeding guidance. The follow-up times and survey contents are as follows:

| Time point | Survey form |
| --- | --- |
| Week 0 | Basic information survey form |
| Week 0, Week 4, Week 8, Week 16 | Follow-up form |
| Week 0,Week 8, Week 16 | Breastfeeding Self-Efficacy Scale - Short Form |
| Week 0 ,Week 8, Week 16 | Epidemiological Depression Self-Rating Scale |

1. **Impact on daily life from participating in this study**

When deciding whether to participate in this study, please carefully consider the potential impact of the above-mentioned surveys and follow-ups on your daily work, family life, etc. Consider the time and transportation issues for each follow-up visit. If you have any questions about the checks and procedures involved in the study, you can consult us.

1. **Risks and adverse reactions/discomfort from participating in this study**

This study requires frequent use of your mobile phone to fill in questionnaires and scales in the WeChat group. If certain questions in the questionnaires and scales make you feel uncomfortable, you may refuse to answer. If you think the frequency or duration of using the mobile phone is too much, you can choose to reduce the usage time or withdraw from the study.

1. **Possible benefits from participating in this study**

Benefits of participating in this study: Direct benefits: Providing the best feeding method, breastfeeding, for the baby, benefiting both the mother and the baby, and reducing the cost of formula milk; Indirect benefits: Obtaining the long-term benefits of breastfeeding.

1. **Are there alternative treatment options if I do not participate in this study?**

You may choose not to participate in this study, and it will not have any adverse effects on your receipt of routine guidance. Currently, hospitals have breastfeeding consultation rooms or pediatric nutrition clinics for issues related to child feeding.

1. **Is it mandatory to participate and complete this study?**

Your participation in this study is entirely voluntary. If you do not wish to participate, you may refuse, and it will have no negative impact on your current or future infant feeding. Even if you agree to participate, you can change your mind at any time and inform the researcher of your withdrawal from the study, which will not affect your receipt of normal medical services. When you decide not to participate in this study, we hope you will inform the researcher in a timely manner, and the researcher can provide suggestions and guidance based on the situation of you and your child.

After you withdraw, no new data related to you will be collected in the future, but the data collected before your withdrawal is also meaningful for this study. The researcher will strictly preserve the information related to your withdrawal from the study until it is finally destroyed. In very rare cases, these data may also be used, for example, when government regulatory authorities conduct supervision, inspection, and statistics, they may request to view all study information, which will include the information related to your participation in the study at that time.

1. **Reasons for terminating your participation in the study**

(1) If the intervention group does not use the “Breastfeeding Aiding Tool” as required for a long time (more than 2 weeks);

(2) Discomfort caused by frequent use of mobile phones;

(3) It is determined that there are situations unsuitable for exclusive/predominant breastfeeding.

In the above cases, the researcher has the right to terminate your participation in the study without your consent.

If this study is terminated prematurely, we will notify you in a timely manner, and your study doctor will provide suggestions for your next treatment plan based on your health condition.

1. **Costs and compensation for participating in this study**

Both the intervention and control groups will receive four free remote or outpatient breastfeeding consultation and guidance opportunities (at enrollment, Week 4, Week 8, and Week 16).

After completing the 16-week follow-up, you will receive a parenting-related book published by the research team.

1. **Handling of research-related injuries**

There are no invasive examinations in this study, and there is no risk of injury.

1. **What do I need to do if I participate in this study?**

Intervention group: Use the “Breastfeeding Aiding Tool” as required

Use WeChat to participate in this study and follow up for 16 weeks.

Provide accurate information for the “Basic Information Survey Form.”

Fill in the “Follow-up Form” four times as required.

Fill in the “Breastfeeding Self-Efficacy Scale - Short Form” and “Epidemiological Depression Self-Rating Scale” three times as required.

Answer phone follow-ups on schedule.

Do not participate in other studies.

Follow the guidance of the study doctor and researchers.

If you have any questions, you can ask at any time.

1. **Will participants' personal information be kept confidential?**

If you decide to participate in this study, your participation and personal information in the study will be kept confidential. Your blood/urine samples will be identified by study number rather than your name. Information that can identify you will not be disclosed to anyone outside the research team unless you give permission. All research members are required to keep your identity confidential. Your files will be kept in a locked cabinet, accessible only to researchers. To ensure that the study is conducted in accordance with regulations, government regulatory authorities or ethics committee members may review your personal information at the research unit as required. When the results of this study are published, no personal information about you will be disclosed.

1. **Who should I contact if I have questions or difficulties?**

If you have any questions related to this study, please contact Researcher Jia Ni, phone number 17611255210. If you have questions related to the rights and interests of the participants, you may contact the Ethics Committee of the Capital Institute of Pediatrics, phone number: 010-85628195.

**Informed Consent Signature Page**

**Participant Declaration**

The researcher has explained to me the background, purpose, procedures, risks, and benefits of the “Randomized Controlled Intervention Study on Promoting Breastfeeding through Mobile Healthcare.” I have had sufficient time and opportunity to ask questions, and the answers provided by the researcher are satisfactory to me. I know whom to contact if I have questions or want to obtain further information. I have read this informed consent form and have decided to participate in this study. I am aware that I can withdraw from this study at any time during the study period without any reason. I have been informed that I will receive a copy of this informed consent form, which includes the signatures of myself and the researcher.

Participant Mother's Name [Regular Script]:

Mother's Signature: Date: Year Month Day

Contact Phone Number (Mobile):

Child's Name [Regular Script]:

Other Guardian's Name [Regular Script]: Relationship to Child:

Other Guardian's Signature: Date: Year/Month/Day

Contact Phone Number (Mobile):

**Researcher Declaration**

I have explained to the participant mother the background, purpose, procedures, risks, and benefits of the “Randomized Controlled Intervention Study on Promoting Breastfeeding through Mobile Healthcare.” I have given her sufficient time to read the informed consent form, discuss with others, and answered her questions about this study. I have informed the participant mother of the contact details for any study-related issues and that she can withdraw from the study at any time during the study period without any reason.

Informed Consent Executor's Signature: Date: Year/Month/Day

Contact Phone Number (Mobile):
