# Supplementary material for: Effectiveness of a Mobile Breastfeeding Monitoring Tool Among Mothers in WeChat Groups on Breastfeeding Exclusivity and Self-Efficacy: Intention-to-Treat and Per-Protocol Analyses of a Randomized Controlled Trial
Source: J Med Internet Res. 2025 Aug 15;27:e67024. doi: 10.2196/67024 (PMC12397754; doi:10.2196/67024)
Supplement: Multimedia Appendix 4 [file jmir_v27i1e67024_app4.docx]

**Multimedia Appendix 4 Baseline characteristics of participants**

| Characteristics | ITT^a^ intervention (n=55) | ITT control (n=54) | χ^2^ (df) | *t* test (df) | *P* value | PP^b^ group using the tool (n=28) | PP group not using the tool (n=81) | χ^2^ (df) | *t* test (df) | *P* value | Completed (n=109) | Dropped out (n=24) | χ^2^ (df) | *t* test (df) | *P* value |
| --- | --- | --- | --- | --- | --- | --- | --- | --- | --- | --- | --- | --- | --- | --- | --- |
| Maternal age(years), mean (SD) | 32.04  (3.32) | 30.66  (3.34) | **-** | 2.14  (105) | *.04^c^* | 32.32  (2.92) | 31.01  (3.49) | - | 1.78  (105) | .08 | 31.36  (3.38) | 30.35  (3.18) | - | 1.14  (122) | .25 |
| Maternal education level, n (%)  High school or below  College  Master or above | 3(5)  28(51)  23(42) | 1(2)  34(63)  17(31) | - | - | .29^d^ | 0(0)  12(43)  16(57) | 4(5)  50(62)  24(30) | - | - | *.04^cd^* | 4(4)  62(57)  40(37) | 1(4)  12(50)  4(17) | - | - | .43 ^d^ |
| C-section, n (%) | 13(24) | 14(26) | 0.1(1) | - | .78 | 4(14) | 23(28) | 2.2(1) | - | .14 | 27(25) | 8(33) | 0.7(1) | - | .39 |
| Sex, male, n (%) | 24(44) | 28(52) | 0.7(1) | - | .39 | 11(39) | 41(51) | 1.1(1) | - | .30 | 52(48) | 6(25) | 4.1(1) | **-** | *.04^c^* |
| Gestational age at birth (weeks), mean (SD) | 39.51  (1.03) | 39.48  (0.92) | - | 0.76  (105) | .88 | 39.64  (1.10) | 39.44  (0.93) | - | 0.35  (105) | .73 | 39.50  (0.98) | 39.00  (2.48) | - | 0.96  (25) ^e^ | .35 |
| Birthweight (g), mean (SD) | 3305.64  (334.78) | 3357.59  (348.23) | - | 0.79  (107) ^e^ | .43 | 3342.86  (320.76) | 3327.41  (349.47) | - | 0.21  (107) | .84 | 3331.38  (340.93) | 3229.17  (365.18) | - | 1.31  (131) | .19 |
| Maternal BMI (kg/m^2^), mean (SD) | 22.98  (2.43) | 23.77  (3.34) | - | 96.72  (107) | .16 | 23.01  (2.48) | 23.50  (3.08) | - | 0.75  (107) | .45 | 23.55  (3.22) | 24.89  (4.41) | - | 1.73  (131) | .09 |
| Diabetes during pregnancy, n (%) | 16(29) | 13(24) | 0.4(1) | - | .55 | 9(32) | 20(25) | 0.6(1) | - | .44 | 29(27) | 5(21) | 0.4(1) | - | .56 |
| Hypertension during pregnancy, n (%) | 3(5) | 3(6) | - | - | 1.00^a^ | 0(0) | 6(7) | - | - | .34 ^d^ | 6(6) | 1(4) | - | - | 1.00 ^d^ |
| Anemia during pregnancy, n (%) | 17(31) | 11(20) | 1.6(1) | - | .21 | 8(29) | 20(25) | 0.2(1) | - | .68 | 28(26) | 9(38) | 1.4(1) |  | .24 |
| Maternal job status, n (%)  Housewife/full maternity leave  Maternity leave and working | 41(74)  14(25) | 42(78)  12(22) | 0.2(1) | - | .69 | 20(71)  8(29) | 63(78)  18(22) | 0.5(1) | - | .50 | 83(76)  26(24) | 18(75)  6(25) | 0.01(1) | - | .9**0** |
| Alcohol, n (%) | 1(2) | 0(0) | - | - | 1.00 ^d^ | 1(4) | 0(0) | - | - | .26 ^d^ | 1(1) | 0(0) | - | - | 1.00 ^d^ |
| Breastfeeding initial time, n (%)  Less than 1 h  1 h-1 day  1 day later | 20(36)  18(33)  17(31) | 22(41)  18(33)  14(26) | 0.4(2) | - | .83 | 10(36)  10(36)  8(29) | 32(40)  26(32)  23(28) | 0.2(2) | - | .92 | 42(38)  36(33)  31(28) | 11(46)  6(35)  7(29) | 0.7(2) | - | .72 |
| Reported breast problems, n (%) | 18(33) | 10(18) | 2.9(1) | - | .09 | 12(43) | 16(20) | 5.8(1) | **-** | *.02 ^c^* | 28(26) | 9(38) | 1.4(1) | - | .24 |
| Reported insufficient breastmilk, n (%) | 9(16) | 13(24) | 1.0(1) | - | .32 | 6(21) | 16(20) | 0.04(1) | - | .85 | 22(20) | 5(21) | - | - | 1.00 ^d^ |
| Reported child with illness, n (%) | 26(47) | 20(37) | 1.2(1) | - | .28 | 14(50) | 32(40) | 0.9(1) | - | .33 | 46(42) | 12(50) | 0.5(1) | - | .49 |

^a^ITT: intention to treat.

^b^PP: per protocol.

^c^Significance of values in italics: Maternal age in ITT (*P*=.04), Maternal education level in PP (*P*=.04), Sex between the dropped-out and completed(*P*=.04), Reported breast problems in PP (*P*=.02).

^d^Fisher exact test.

^e^Unequal variances.
