# Supplementary material for: Effectiveness of a Mobile Breastfeeding Monitoring Tool Among Mothers in WeChat Groups on Breastfeeding Exclusivity and Self-Efficacy: Intention-to-Treat and Per-Protocol Analyses of a Randomized Controlled Trial
Source: J Med Internet Res. 2025 Aug 15;27:e67024. doi: 10.2196/67024 (PMC12397754; doi:10.2196/67024)
Supplement: Multimedia Appendix 5 [file jmir_v27i1e67024_app5.docx]

**Multimedia Appendix 5 Qualitative feedback information**

| **Positive feedback** | **Negative feedback** |
| --- | --- |
| 1. It has accurately/fast features (functions) and components (buttons/menus) work. 2. It has necessary links between screens. 3. It is content correct, well written, and relevant to the goal/topic. 4. The arrangement and size of buttons, icons, menus and content on the screen are appropriate. 5. It has good quality/resolution of graphics used for buttons, icons, menus and content. 6. The information within the app is comprehensive but concise. 7. The information has a credible source. 8. It is easy to learn how to use. 9. The functions meet the needs for this type of application. | 1. Mothers couldn’t customize the settings and preferences that mothers would like to (e.g. sound, content and notifications). 2. Mothers couldn’t input, provide feedback, contain prompts (reminders, sharing options, notifications, etc.). 3. Mothers prefer paper-based breastfeeding records. 4. Mothers prefer other breastfeeding apps. 5. It is too troublesome to record breastfeeding and mothers have forgotten to use the app. |
